# Salient Conclusive Remarks on Epidemiology and Clinical Manifestations of Pediatric COVID-19: Narrative Review

**DOI:** 10.3389/fped.2020.584694

**Published:** 2020-12-01

**Authors:** Abdelwahid Saeed Ali, Ahmed Mossa Al-Hakami, Ayed Abdullah Shati, Ali Alsuheel Asseri, Saleh Mohammed Al-Qahatani

**Affiliations:** ^1^Department of Microbiology and Clinical Parasitology, College of Medicine, King Khalid University, Abha, Saudi Arabia; ^2^Department of Child Health, College of Medicine, King Khalid University, Abha, Saudi Arabia

**Keywords:** COVID-19, SARS-CoV-2, pediatrics, epidemiology, symptoms

## Abstract

The ongoing pandemic of COVID-19, which is caused by the novel coronavirus named severe acute respiratory syndrome coronavirus 2 (SARS-CoV-2), constituted significant public health concerns and impacted the human populations with massive economic and social burdens worldwide. The disease is known to infect people of all ages, including children, adults, and the elderly. Although several reports about pediatric COVID-19 were seen in the literature, we believe that the epidemiology and pathology of the infection described in these reports are not conclusive. Therefore, in this scientific communication, a narrative review study was performed to shed some light on the characteristic epidemiological features and clinical phenotypes of pediatric COVID-19. In this report, we had compiled and presented the different epidemiological features of the disease related to the age of infection, virus acquisition, explanations of the low infectivity rates, and consequences of infections. The discriminatory clinical manifestations of the disease in children were also addressed and discussed in this review. The search included the data published from the date of the start of the pandemic in December 2019 up to October 2020. Our literature search revealed that children of all ages, including neonates, had been infected by the virus. Despite the fact that pediatric COVID-19 is less common to occur, as compared to the disease in adults, the infected children usually manifest the disease symptomatology in benign form. Asymptomatic and symptomatic adult patients are the primary source of the virus to the children. Intrauterine transmission of the virus and breastfeeding infections to the neonates were hypothesized in some studies but ruled out since they were not confirmed. Intensive review and discussion warranting the low infection rates and benign conditions of COVID-19 in children were also made in this study. As documented in many studies, the infectivity, morbidity, and mortality rates of the disease among the children populations are much lower than those in adults. They also seem to be lower than those observed during SARS-CoV and MERS-CoV epidemics. The described clinical phenotypes of COVID-19 in children do not differ much from those of adults, and complications of the disease seem to be associated with comorbidities.

## Introduction

Human coronaviruses (HCoVs) are pathogens responsible for respiratory and enteric infections (1). Formerly, four HCoVs were well known to cause mild respiratory infection. However, in the last two decades, an additional three highly morbid and fatal HCoVs have emerged. They included the severe acute respiratory syndrome coronavirus (SARS-CoV) (2, 3) and the Middle East respiratory syndrome coronavirus (MERS-CoV) (4, 5). The severe acute respiratory syndrome coronavirus virus 2 (SARS-CoV-2) is the third HCoV, which recently emerged in late December of 2019, causing the severe respiratory illness termed COVID-19 (6–11). The virus was confirmed to belong to the beta-coronavirus group (12, 13). This virus originated from Wuhan, the capital city of Hubei province in China, and the identified source of the virus was some wild animals (14, 15). The virus firstly caused an epidemic of the disease in China and then spread throughout the world, resulting in a global pandemic characterized by high infectivity and mortality rates (16–22). The rapid virus spread from one country to another was attributed to the enormous human movements throughout the globe (9, 23). The prominent clinical manifestations of COVID-19 among adult patients mentioned in many reports included fever, dry cough, dyspnea, myalgia, fatigue, and pneumonia (9, 24–26). In advanced and critical cases, symptoms of dizziness, generalized weakness, vomiting, and diarrhea were also observed (27). Deaths among COVID-19-infected patients were observed to be directly correlated with acute respiratory distress syndrome (ARDS) (23). In children, some studies suggested that the symptomatology of COVID-19 does not look much different from that of adults. However, other studies indicated that the clinical manifestations of pediatric COVID-19 widely differ from that of adults (28).

COVID-19 was proved to affect all age groups of people, including newborns, infants, children, adults, and the elderly. Primarily, it was mentioned that children are always asymptomatic to COVID-19, then shortly after, there were many reports indicated that they can develop the pathology and symptomatology of the disease (11, 29). The results obtained in several epidemiological studies showed that the disease is more severe and fatal among the elderly individuals (30), whereas the infectivity rates and the fatalities are lower among the children populations (23, 31).

In the light of all these data, we would like to summarize in this report the significant findings related to the characteristic epidemiological features and clinical aspects of pediatric COVID-19. In this review, we would also like to cast some light on the medical challenges associated with the clinical management of the disease among children worldwide.

## Methods

### Search Strategy

A comprehensive electronic search in the literature for the characteristic epidemiological and clinical features of pediatric COIVD19 was done in this study. The targeted information was obtained from the data reported from different regions and countries of the world. The search had been done in PubMed, MEDLINE, and Google Scholar databases. The keywords used in the search were COVID-19, SARS-CoV-2, novel coronavirus, pediatric, neonates, infants, children, epidemiology, and symptoms.

### The Selection Criteria

The articles published within the year 2020 were considered in the study. The other inclusion criteria for the search is the confirmed cases of COVID-19 among pediatric patients using the reverse-transcription polymerase chain reaction (RT-PCR). Articles published only in the peer-reviewed journals were considered. Additionally, some of the considered articles were further used for the search for more relevant publications. Any article considered relevant and appropriate for the topic of this study was carefully read and summarized before the extracted data used in the description.

### Retrieval and Use of the Data

From any of the selected articles, the significant elements of the epidemiology of the disease in children, including the age of infection; mean infection, incidence, and prevalence rates; morbidities; and fatalities, were extracted and recorded. The characteristic clinical features described were also recorded. The medical interventions and management practices employed were also recorded if applicable. Then, a narrative description was generated from the search outcome and presented in this manuscript.

### Epidemiology

The epidemiological characteristics of the COVID-19 infections in children were addressed in this review to discuss and unravel the following elements: age of infections, infection acquisition, incidences, prevalence, morbidities, and mortalities. Generally, the major epidemiological features of pediatric COVID-19 are demonstrated in Table 1.

**Table 1:** Major distinctive epidemiological and clinical features between pediatric and adult COVID-19.

	**Pediatric COVID-19**	**Adult COVID-19**	**References**
**Epidemiology**			
Age of infection	Children of all ages can be infected with SARS-COV-2, including neonates.	All age groups of adults can be infected with SARS-COV-2 and develop severe COVID-19	(32–36)
Infection acquisition	* Children acquire the infection through direct contact with the other symptomatic or asymptomatic patients. * Perinatal infections with the virus to babies born from positive pregnant mothers did not occur. * Vertical and breastfeeding transmissions were not confirmed. * Familial clusters infection is the dominant transmission mean for pediatric patients.	* Direct contact with infected individuals shedding the virus as aerosols in their sections. * Contaminated surfaces with the secreted droplets from infected individuals constitute great hazards as sources of infection.	(37–43)
Infectivity rates	Incidence rates ranging from 1 to 2% were recognized among the pediatric populations.	* Higher incidence rates among adult populations were always recognized.	(44–46)
Morbidities and mortalities	* Morbidities among pediatric groups of patients are very low as compared to adults. * Fatalities due to COVID-19 account for about 0.9–3% in children.	* Morbidities among adults population is about 15%. * Mortalities were estimated to be about 5%. * The case fatality rate was estimated as 2–3%.	(38, 45, 47–50)
**Clinical aspects**			
Incubation period (I.P)	* Some studies estimated the I.P of COVID-19 as 5–6 days; other studies estimated it as 9-11 days.	* The median I.P for adults infected with COVID-19 was estimated to be 5.1 to 11.5.	(9, 43, 51–54)
Disease severity	* Mild infections, benign disease and reduced complications of COVID-19 in pediatrics were observed.	* The disease conditions vary between mild, moderately ill, severe, and critical infections.	(52, 55–58)
Fever and respiratory symptoms	Fever and respiratory symptoms are common among pediatric COVID-19 patients but are not considered hallmark or discriminatory diagnostic features.	* Fever and upper respiratory tract symptoms were discussed in many studies as primary factors suggestive for COVID-19 in adults.	(28, 29, 58, 59)
ARDS	Pneumonic pediatric cases are uncommon. In critical conditions, ARDS serves as a serious complication resulting in respiratory failure.	* ARDS is common mainly among the critically ill patients and those suffering from other underlying respiratory or cardiac conditions.	(50, 59, 60)
Biochemical findings	CK-MB, CRP, PCT, and LDH were elevated on frequent bases among pediatric COVID-19 patients.	Changes in the biochemical markers and inflammatory variables among adult patients were observed. In asymptomatic patients, these changes are minimal.	(36, 61, 62)
Histopathology	Features included ground-glass opacities and patchy shadows in the lungs were reported in severe and critical cases.	Bilateral or unilateral ground-glass opacities in the lungs were observed. They mainly involved the right lower lobes.	(27, 28, 63)

*COVID-19, coronavirus disease 19; ARDS, acute respiratory distress syndrome; I.P, incubation period; CK-MB, creatine kinase-MB; CRP, C-reactive protein; PCT, procalcitonin; LDH, lactate dehydrogenase*.

#### Age of Infection

It has been confirmed that neonates can be infected with COVID-19, although few cases were reported in some studies (32). A controversial discussion about the transmission of the virus to the neonates was made in the literature. Although the vertical transmission of SARS-CoV-2 during pregnancy was assumed and discussed in some neonatal COVID-19 cases, a concrete evidence for the intrauterine transmission of the virus from the pregnant mother to the fetus does not exist to date (64). The maternal milk as a source of infection of newborns with COVID-19 had also been hypothesized in some studies, but it had confirmed that maternal milk could not serve as a vehicle for the virus to babies. It is, therefore, recommended that breastfeeding for babies from the infected mothers can be continued without risk to them (65). The known fact is that neonates get infected by the virus through close contact with the virus-infected patients or carriers such as family members and healthcare givers (66).

The first confirmed reported case of COVID-19 in infants is a 3-month-old female from Xiaogan, Hubei province of China (33). In this report, the patient was tested positive for the virus using the gold standard RT-PCR test. She was presented with transient fever and showed glass appearance opacity in the lung during the chest CT scan radiography. This infant recovered shortly after the test. This report substantiated that infants can be infected with the virus but mildly and recover faster than older children. Later, many epidemiological and clinical observations for the disease in infancy were reported (34).

The first reported case of COVID-19 in children worldwide was observed in a 7-year-old child in Chongqing city of China. This case is considered the first that indicated the possibility of children being infected with SARS-CoV-2 (35). During the early days of the disease outbreak, there was an uncertain notion that children show milder symptoms of the disease than adults. After that, it becomes a kind of credo to the scientific arena and the public as well. In a comprehensive review article recently published by Brandon and coworkers, they had concluded that children of all ages are vulnerable to COVID-19 infection with low infectivity rates and less aggressive clinical consequences (36). Additionally, a recent nationwide case series screening for the definite pediatric COVID-19 cases in some Chinese communities had also confirmed that children of all ages are vulnerable to the disease and with no significant variations among the genders (37).

#### Infection Acquisition

There are many reports in the literature discussing the source and means of transmission of SARS-CoV-2 to the children. Many of these reports indicated that the primary source of infection of children with the virus is the other symptomatic or asymptomatic patients (37–39). The symptomatic patients were known to serve as a potential source of the virus to the children during and after the incubation period of the disease (39). As for the newborns, an interesting study showed that the contamination of mother's milk with SARS-CoV-2 through the touch of infected mothers' hands or pumps could be an effective means of virus transmission (67). This study also concluded that the feeding containers contaminated with the virus could also be a powerful tool for virus transmission to babies.

Although the perinatal infection of the newborns born to positive mothers with COVID-19 was assumed during the onset of the pandemic, it had been confirmed in some studies that it is unlikely to occur if they are properly and adequately managed during vaginal delivery (40, 68). In another study deliberately made to unveil the COVID-19 possible sources of infection to children, it had been mentioned that the familial clusters including infected individuals, asymptomatic or ill, constitute a substantial challenge of infection with the virus (41). Additionally, in the same context of this familial infection, other studies also confirmed that children are more likely to contract the virus through household contacts (42). Although limited data in the literature regarding the vertical transmission of the SARS-CoV-2 from infected pregnant mother to the fetus are available, some epidemiological studies in this context concluded that this means of transmission is unlikely to exist (42, 43). As per the virus source to the children, another comprehensive study included a considerable number of Chinese children with definite COVID-19 indicated that most of them acquired the infection from the familial sources (69). As a general important epidemiological note, the sources and transmission modes of the virus for pediatric COVID-19 is considered extremely important for understanding the role of children in the dynamics of the pandemic as they may become a sizable factor in the disease spread in many parts of the world (70).

#### Infectivity Rates

The incidence and prevalence rates of COVID-19 among children populations in many parts of the world were firstly assumed and declared concomitant with those of the SARS-CoV-1 and MERS-CoV infections when their epidemics occurred in 2002 and 2012, respectively. In these two epidemics, the incidences showed extremely lower levels among children as compared to adults. However, as the COVID-19 pandemic progresses and due to the rapid spread of the disease throughout the globe, another theory saying that the incidence rate of the disease among children is higher than those of previously mentioned epidemics has emerged. Our search in the literature revealed that different infectivity rates of pediatric COVID-19 were reported in different parts of the world. The infectivity rates of COVID-19 among the children populations were found to be correlated with those of the disease in adults, the various control measures adopted, and the general capacity of each state to control the infection. A Chinese study including laboratory-confirmed cases of the disease from across the country reported an incidence rate of <1% among the children below 10 years of age (44). This percentage was observed to increase in children older than this age. Another significant conclusion derived from this study is the increase in the number of pediatric COVID-19 cases with an increase in the number of adult cases. In another study also addressing the correlation between the age of children and infectivity rates of the SARS-CoV-2 in the communities, it was observed that out of the total population tested positive for the virus, about 2% of them were children who are <19 years old (45). As regards neonatal COVID-19, in a recent study conducted among infected pregnant mothers to elucidate the possible means of transmission and infectivity rates, some researchers confirmed that the disease is less common to occur. They also assured that the disease is asymptomatic among the neonates born to these mothers (71). These findings indicated that the babies born from mothers infected with the virus can still be safe from being infected with the virus even if they are vaginally delivered or breastfed on a condition they should be entirely managed.

As a general conclusion, after an intensive literature search, we can mention that SARS-CoV-2 is infectious to children of all ages but with low susceptibility rates, benign clinical outputs, and minor aggressiveness.

#### The Explanation for Low Infectivity Rates and Benign Pediatric COVID-19

A significant concern and rigorous disputes were raised by scientists, clinicians, and epidemiologists to give plausible explanations for the well-established fact that pediatric COVID-19 is characterized by low infectivity rates, mild infections, and reduced complications, as compared to the disease in adult populations in the various communities. Some clinicians suggested that the infectivity rates are lower among children as they are less likely to undergo immunosuppressant therapies throughout their lives. On the contrary, that is much likely for adults who are mostly suffering from diseases treated with such kinds of drugs as anti-rheumatics (72).

To explain these epidemiologic and clinical patterns of pediatric COVID-19 from an immunological perspective, some researchers referred to the idea of the immune memory generated from previous exposure to antigens, including both specific antigens exciting the specific immunity and preserved antigens stimulating the non-specific immune responses. They think that these immune responses might assist in fighting off COVID-19 (73, 74). Additionally, these researchers also referred to the fact that the environmental factors that influence and modulate the immune system are mostly identified in the early days of human life (73). Therefore, the low infectivity rates of COVID-19 among the pediatric populations can be attributed to these effects. Some immunologists also believe in the thymus, one of the principal and essential immune organs that is much active in childhood than in adulthood (74). This is an exciting kind of justification but remains a controversial point of explanation as no proof exists for it. In other studies, some scientists assumed that children might express more powerful innate immune reactions to the virus and hence show less aggressive infection (29, 75). Another immunology explanation is the notion that kids are more likely exposed to the other mildly infectious types of HCoVs, and they developed immunity against these viruses. This immunity might be partially protective against COVID-19. It had also been assumed that children are frequently exposed to other pathogens in areas like school environments and producing antibodies that are variably required and sufficient to protect them against COVID-19 (76). Although the immune reactions to viral pathogens are highly specific, some researchers assumed that cross-protection reactivity between viruses causing respiratory infections in children, including rhinoviruses, respiratory syncytial virus (RSV), and influenza with SARS-CoV-2, might exist, resulting in milder infections among them (75, 76). In previous similar studies, it had also been reported that children are less vulnerable to severe and fatal viral infections, like chickenpox and measles, as compared to adults since the natural immune and defense components of the body are vital in childhood and decline with increasing age (77). This effect might also be the case with COVID-19.

Utilizing the basic biological properties of the causative virus of COVID-19, virologists had also explained the low infectivity rates of the disease among children. They understand that SARS-CoV-2 utilizes the angiotensin-converting enzyme 2 (ACE2) molecules on the host cell membranes as receptors to enter the cells. It was also well-known that children express higher ACE levels and lower levels of ACE2 in their sera, with the consideration of the antagonistic relationship between ACE and ACE2. The levels of these molecules are almost different in adults (78, 79). We believe that this issue concerning the low expression, maturation, and distribution of the receptors to the virus represents the principal factor for warranting the low infection rates and mild disease expression in children over adults.

To warrant the decreased severity of the disease and low risk of complication development in children infected by SARS-CoV-2, epidemiologists attributed that to the absence or the less frequent occurrence of other underlying chronic conditions in children, such as cardiovascular diseases and diabetes, as they are in adults. Other scientists justified the small proportions of infections with COVID-19 among children worldwide with the few numbers of tests carried out for them in a particular population. The children are more likely to develop milder or asymptomatic forms of the disease, and hence many pediatric cases can be missed diagnosis of the virus infection in the specific community (43). From a behavioral and social perspective, some scientists had emphasized that children have low susceptibility to the disease as they had more home confinement and thus lower possibility of exposure to the virus compared to adults (29, 80). We cannot generate a statement of consensus for this type of explanation as the issue of children's frequency of exposure to the virus is different between the communities. Here, we also strongly think that the issue is highly dependent on the children's education methods adopted during the pandemic period, and if they attend school through physical attendance or online learning. The commonly circulating explanation in the literature and the media for the mild disease and fast recovery of children from COVID-19 is the common social behaviors of children, including non-smoking and less exposure to air pollution.

#### Morbidities and Mortalities

The morbidity, mortality, and case fatality rates (CFRs) of pediatric COVID-19 were estimated and discussed in many reports in the literature. There are apparent variations observed between the findings of the studies as per these epidemiological correlates. In a recent study carried out to determine the frequency of the early onset of neonatal COVID-19 among neonates born to mothers with definite COVID-19, significant results had been obtained showing the low morbidity rate, and no deaths were reported among the neonates (81). As a conclusive remark, COVID-19 had low morbidity and mortality rates among children of all ages. Similar trends of low morbidities and mortalities were also reported among pediatric patients during the outbreaks of SARS-CoV 1 (82) and MERS-CoV (83). Some other reports indicated that the low morbidities and mortalities for pediatric COVID-19 during the pandemic in many parts of the world were also noticed during our search in the literature (47, 48). The CFR of COVID-19 among children was also determined and compared with those of SARS-CoV-1 and MERS-CoV. While CFR ranges between 6 and 17% for SARS-CoV (84, 85) and between 20 and 40% for MERS-CoV (86–88), it had been reported in some studies to range between 0.9 and 3% for SARS-CoV-2 (38, 45). This finding indicates that COVID-19 is less serious to children compared to SARS and MERS HCoVs. Among the neonates, the disease conditions were also proven mild, with many minor morbidities and mortalities detected (49). The mortality rates of COVID-19 among the pediatric patients correlated with the age of children in the total population; that is, lower rates were observed among the younger children (44). As obesity was suggested as a risk factor to increase the vulnerability of adults to severe and fatal viral infections (89), we also think that obesity may constitute an important factor for deteriorating the conditions of clinically COVID-19-infected children.

It is pretty accurate that the virus is less harmful to children as discussed in many studies; however, from an essential epidemiological viewpoint, children may also serve as facilitators for the viral transmission in the community, and this necessitates some measures to be implemented, including their non-interaction with the elderly and individuals who are likely to develop severe COVID-19 infections (90).

### Clinical Manifestations

The major distinctive clinical features between pediatric and adult COVID-19 are presented in Table 1. The incubation period of COVID-19 in pediatric patients was appraised and discussed in several studies. It had been estimated as 5–6 days in some reports (43, 51) and 9–11 days in others (9, 52, 69). An overwhelming data retrieved from the literature revealed that the asymptomatic nature of COVID-19 among many infected children worldwide was observed (55–57). These asymptomatic cases were mainly observed among children who do not suffer other underlying health problems, and the recovery was reported to occur within a couple of weeks (52, 55). Symptomatic cases of COVID-19 in children vary depending on the disease severity, ranging from mild, moderate, severe, and critical (28, 37, 69).

Neonatal COVID-19 infections are characterized by sudden onset with non-specific clinical features (66). Temperature instability, poor feeding, hypoactivity, and tachypnea are very suggestive clinical features for COVID-19 infection in the neonates (28). It had also been concluded in some studies that symptoms of COVID-19 in newborns are uncommon and mild, and complications are unlikely to occur if they are correctly and adequately managed (33).

In children, fever, dry cough, elevation in the respiratory and heart rates, dyspnea, and sometimes nasal obstruction are clinical indications of the disease in mild cases. Diarrhea can also be detected in some advanced cases (35). Pneumonic pediatric cases due to COVID-19 were also observed in some studies though uncommon (28). A severe and fatal childhood COVID-19 pneumonia was reported for the first time in February 2020, as described by Chen et al. (57). In critically ill pediatric patients, serious complications such as septic shock and ARDS resulting in respiratory failure were also observed (29, 59, 60). In immunocompromised children and those suffering from other respiratory or heart diseases, more complicated COVID-19 conditions were evident (56).

Although respiratory symptoms and fever are the major discriminatory clinical features in adults, they are not considered the hallmark for COVID-19 among pediatric patients (28). Another exciting clinical feature that distinguishes between adult and pediatric COVID-19 is that, in children, the disease is unlikely to progress to severe respiratory conditions necessitating ICU admission (24, 91).

Despite the intensive search made in the literature for the pathophysiology of COVID-19 in children, little was noted about the laboratory abnormalities of the disease. Inconsistency in the leucocyte indices was detected among pediatric COVID-19 patients. Therefore, the leucocyte counts should not be considered as markers for disease occurrence or severity. Creatine kinase-MB (CK-MB), C-reactive protein (CRP), procalcitonin (PCT), and lactate dehydrogenase (LDH) were elevated on frequent bases during the infection (36, 61). As regards histopathology associated with COVID-19 in pediatric patients, features including ground-glass opacities and patchy shadows in the lungs were reported in severe and critical cases (28, 63). Lesions in the subpleural areas in the severe cases of the disease were also observed among the infected children when the chest CT scan was employed (92). These findings promoted the CT scan as a potential diagnostic tool for the detection and characterization of COVID-19 pneumonia in pediatric medicine. These pathological findings can also be utilized to provide a concrete basis for the differentiation between pediatric and adult COVID-19 infection.

In contrast to adults, favorable consequences and prognosis of COVID-19 in children are concluded in many studies as milder clinical presentations and smaller numbers of deaths were reported (58, 93).

## Conclusions

Following the intensive literature search employed in this study, some conclusive remarks as per the characteristic epidemiological features and clinical patterns of COVID-19 among pediatric patients during this ongoing pandemic can be highlighted:

- Among children, there is no age specificity for the virus. Children of all ages, including newborns, had been infected by SARS-CoV-2.
- Symptomatic and asymptomatic patients constitute the primary source of COVID-19 to children. Direct and close contacts with these patients represent the primary means of virus transmission. Intrauterine transmission of the virus to babies of pregnant women was not confirmed. Breastfeeding was also not proven as a means of infecting neonates.
- Low infectivity, morbidity, mortality, and CFRs, and benign disease were known to be related to pediatric COVID-19 compared to the disease in adults. These findings were explained from virological, immunological, epidemiological, clinical, and behavioral perspectives in this review report.
- Despite the low infectivity rates and mild clinical manifestations observed among pediatric COVID-19 patients, children may represent a potential source of the virus for adults in the communities.
- Clinical presentations of COVID-19 in children widely differ from those in adults. Fever and respiratory symptoms are not discriminatory or hallmark in definite pediatric cases.
- Both symptomatic and asymptomatic cases of the disease in children were reported. Symptomatic cases vary between mild, moderate, severe, and critical conditions depending on immunosuppression and other chronic respiratory and cardiac diseases.
- As reported in many parts of the world, children do not always develop severe and fatal COVID-19, and hospitalizations are less common as in adult cases.
- Favorable prognosis and consequences of COVID-19 infection in children were known as compared to the situation in adults.

## Author Contributions

All authors listed have made a substantial, direct and intellectual contribution to the work, and approved it for publication.

## Conflict of Interest

The authors declare that the research was conducted in the absence of any commercial or financial relationships that could be construed as a potential conflict of interest.
